# Quality Improvements in Management of Children with Acute Diarrhea Using a Multiplex-PCR-Based Gastrointestinal Pathogen Panel

**DOI:** 10.3390/diagnostics11071175

**Published:** 2021-06-28

**Authors:** In Hyuk Yoo, Hyun Mi Kang, Woosuk Suh, Hanwool Cho, In Young Yoo, Sung Jin Jo, Yeon Joon Park, Dae Chul Jeong

**Affiliations:** 1 Department of Pediatrics, College of Medicine, The Catholic University of Korea, Seoul 06591, Korea; yoohymn@naver.com; 2 Uijeongbu Eulji Medical Center, Department of Pediatrics, Eulji University School of Medicine, Seongnam 11759, Korea; wssuh82@gmail.com; 3 Department of Laboratory Medicine, College of Medicine, The Catholic University of Korea, Seoul 06591, Korea; hanwool.cho@catholic.ac.kr (H.C.); yiy00@naver.com (I.Y.Y.); 1100379@cmcnu.or.kr (S.J.J.); yjpk@catholic.ac.kr (Y.J.P.)

**Keywords:** culture, diagnosis, diarrhea, infections, polymerase chain reaction

## Abstract

Conventional methods for etiologic diagnoses of acute gastroenteritis (AGE) are time consuming and have low positive yield leading to limited clinical value. This study aimed to investigate quality improvements in patient management, antibiotic stewardship, and in-hospital infection transmission prevention using BioFire^®^ FilmArray^®^ Gastrointestinal Panel (GI Panel) in children with acute diarrhea. This was a prospective study recruiting children < 19 years old with new onset diarrhea during the study period, and a matched historical cohort study of children diagnosed with AGE during the 4 years prior. Patients in the prospective cohort underwent stool testing with GI Panel and conventional methods. A total of 182 patients were included in the prospective cohort, of which 85.7% (n = 156) had community-onset and 14.3% (n = 26) had hospital-onset diarrhea. A higher pathogen positivity rate for community-onset diarrhea was observed by the GI Panel (58.3%, n = 91) compared to conventional studies (42.3%, n = 66) (*p* = 0.005) and historical cohort (31.4%, n = 49) (*p* < 0.001). The stool tests reporting time after admission was 25 (interquartile range, IQR 17–46) hours for the GI Panel, and 72 (IQR 48–96) hours for the historical cohort (*p* < 0.001). A significant reduction in antibiotic use was observed in the prospective cohort compared to historical cohort, 35.3% vs. 71.8%; *p* < 0.001), respectively. Compared to the GI Panel, norovirus ICT was only able to detect 4/11 (36.4%) patients with hospital-onset and 14/27 (51.8%) patients with community-onset diarrhea. The high positivity rate and rapid reporting time of the GI Panel had clinical benefits for children admitted for acute diarrhea, especially by reducing antibiotic use and enabling early adequate infection precaution and isolation.

## 1. Introduction

Acute gastroenteritis (AGE) remains a common cause of morbidity and mortality in infants and children worldwide [1,2,3]. Although global mortality from diarrheal diseases have declined significantly over the past two decades, diarrhea is still the fifth leading cause of death in children under 5 years of age, and AGE is one of the common causes of mortality in children in low-income countries [2]. Even in developed countries, AGE is a major causes of emergency room visits or hospitalizations in children, and the resulting health care and economic burden of AGE remains high [4,5].

AGE is usually caused by infections with viral, bacterial, or parasitic pathogens [6]. Differential diagnoses of the causative pathogens based on clinical symptoms may be difficult due to similar presentations. However, depending on the cause, decisions on treatment, isolation, follow-up care, and further investigations may vary [5,7,8,9]. Most children with AGE recover spontaneously with proper rehydration and nutrition [9]. However, depending on the causative pathogen in addition to the clinical course of AGE, antibiotic treatment, active infection control, or close monitoring of possible complications, such as hemolytic uremic syndrome, may be required [7,10,11].

Conventional methods for etiologic diagnoses of AGE include culture for bacteria, immunoassays for viruses, and microscopy or enzyme immunoassays for parasites [12,13]. Unfortunately, these tests are time consuming have low positive yield. Therefore, patients may be unable to receive proper treatment or isolation precaution in a timely manner or may receive unnecessary intervention. In order to overcome the limitations that conventional methods have, the use of multiplex-PCR-based gastrointestinal (GI) pathogen panels is increasing [14,15].

The BioFire^®^ FilmArray^®^ Gastrointestinal Panel (GI Panel) received FDA approval in 2014 and can identify 22 of the most common GI pathogens, including bacteria, parasites, and viruses, directly from stool samples within approximately one hour [16]. If this test allows rapid and accurate pathogen identification in children with diarrhea, it can be an important tool that overcomes the current etiologic diagnostic dilemmas in pediatric AGE.

Currently, there is still insufficient research on whether multiplex-PCR-based GI pathogen panel examination can lead to quantifiable improvements in patient care, and no studies have been conducted specifically on children [17,18,19,20]. Therefore, the primary endpoint of this study was to investigate whether using the BioFire^®^ FilmArray^®^ Gastrointestinal Panel (GI Panel) in children with acute diarrhea brought on quality improvement in patient management, antibiotic stewardship, and in-hospital infection transmission prevention.

## 2. Materials and Methods

### 2.1. Study Design and Participants

This study was carried out in two parts: a prospective study recruiting children with new onset diarrhea during the prospective study period, and a retrospective cohort study of children diagnosed with acute infectious diarrhea during the 4 years prior to the prospective study period.

### 2.2. Prospective Stool Collection Design

The first part of the study was a prospective study recruiting children below 19 years old that fit one of the two following criteria: (1) visited the emergency department or were admitted with symptoms of acute diarrhea with diarrhea onset within 72 h (community-onset diarrhea), or (2) patients that had no diarrhea at admission, and a new onset of diarrhea at least 72 h after admission for the purpose of treating another disease (hospital-onset diarrhea). Patients were excluded if they had an onset of diarrhea >72 h before stool sample collection or chronic diarrhea.

#### 2.2.1. BioFire^®^ FilmArray^®^ Gastrointestinal Panel

Patients with acute diarrhea that were clinically diagnosed with AGE of infectious origin and that also agreed to participate in this study were given an additional stool sample collection container other than the ones given for the routine evaluation of the causative pathogen of acute diarrhea (Supplement Table S1). When the stool was collected and sent to the laboratory, the stool specimen was immediately transferred to a Cary Blair transport medium (Faecal swab; Copan, Brescia, Italy). The specimen then underwent BioFire^®^ GI Panel (BioFire Diagnostics, LLC, Utah Salt Lake City, UT, USA; bioMérieux, Marcey-l’Etoile, France) via FilmArray^®^ 2.0 platform according to the instructions of the manufacturer.

The BioFire^®^ GI Panel is able to detect the following: adenovirus (AdV) F40/41, astrovirus (AstV), norovirus (NoV) genogroup GI/GII, rotavirus (RV) group A, sapovirus (SaV) genogroups GI, GII, GIV, and GV, *Campylobacter* (*C. jejuni*/*C. coli*/*C. upsaliensis*), toxigenic *Clostridioides difficile*, *Plesiomonas shigelloides*, *Salmonella* spp., *Vibrio* (*V. parahaemolyticus*/*V. vulnificus*/*V. cholerae*; with specific detection of *V. cholerae*), *Yersinia enterocolitica*, enterotoxigenic *Escherichia coli* (ETEC), enteropathogenic *E. coli* (EPEC), Shiga toxin-producing *E. coli* (STEC, with specific detection of *E. coli* O157), *Shigella*/enteroinvasive *E. coli* (EIEC), enteroaggregative *E. coli* (EAEC), *Cryptosporidium*, *Cyclospora cayetanensis*, *Entamoeba histolytica*, and *Giardia intestinalis*. For each specimen, the software’s run-time to generate BioFire^®^ GI Panel results is one hour. The results were then texted to the physician of the patient within 4 working hours of stool submission. Depending on the results of the BioFire^®^ GI Panel or stool tests, the patient’s management was altered (Supplementary Table S1). The time taken for the physician to receive the reports of the BioFire^®^ GI Panel after admission was recorded for each case.

#### 2.2.2. Routine Stool Pathogen Detection in the Prospective Cohort

Each patient in the prospective cohort underwent the following routine conventional stool pathogen detection tests with the stool specimen collected at the same stool passage as the specimen obtained for BioFire^®^ GI Panel: (1) traditional bacterial stool culture with species identification by Vitek^®^ MS (bioMérieux, Marcy L’Etoile, France) or Vitek^®^ 2 (bioMérieux), (2) AllplexTM GI-Bacteria(I) assay (Seegene Inc., Seoul, Korea) that is able to detect *Salmonella* spp., *Campylobacter* spp., *Clostridium difficile* Toxin B, *Shigella* spp./EIEC (ipaH), *Vibrio* spp., *Yersinia enterocolitica*, and *Aeromonas* spp., (3) Xpert^®^ *C. difficile* assay (Cepheid, Sunnyvale, CA, USA), (4) conventional PCR for diarrheagenic *E. coli* as previously described [21] and (5) Immunochromatography test (ICT) careUS^TM^ Rotavirus Plus (Wells bio, Seoul, Korea) for rotavirus and CerTest Norovirus (Biotec S.L, Zaragoza, Spain) for norovirus.

### 2.3. Retrospective Historical Cohort Study

The second part of the study was undertaken in a historical cohort of patients diagnosed with ‘infectious enterocolitis’ or ‘acute gastroenteritis’ with a community-onset of diarrhea within 72-h of stool sample collection for etiologic diagnosis. All patients below 19 years old that were admitted at Seoul St. Mary’s hospital during a 4-year period prior to the prospective study period were reviewed.

For each patient in the community-onset prospective cohort, patients in the historical cohort were matched using two fixed variables to match the two cohorts: age and season of diagnosis (Spring, March to May; Summer, June to August; Fall, September to November; Winter, December to February). One patient from the allocated historical cohort was randomly selected for each patient in the prospective cohort, yielding a 1:1 (prospective: historical) variable matching ratio.

#### Stool Pathogen Detection in the Historical Cohort

The following tests were available for the detection of stool pathogens in the historical cohort: (1) traditional bacterial stool culture with species identification by Vitek^®^ MS (bioMérieux, Marcy L’Etoile, France) or Vitek^®^ 2 (bioMérieux), (2) conventional PCR for Salmonella species, (3) Clostridium difficile Toxin gene PCR, (4) conventional PCR for diarrheagenic *E. coli* as previously described [21] and (5) Immunochromatography test (ICT) careUS^TM^ Rotavirus Plus (Wells bio, Seoul, Korea) for rotavirus and CerTest Norovirus (Biotec S.L, Zaragoza, Spain) for norovirus.

### 2.4. Clinical Data Collection

For patients included in the prospective study, the following clinical parameters were collected during the study: birthdate, sex, symptom onset date, admission date, discharge date, initially administered drugs, changes in drugs or treatment plan, stool etiologic test results, time to etiologic diagnosis, type of infection precaution applied, and application date. For patients in the historical cohort, their electronic medical records were retrospectively reviewed for the clinical parameters. Study participants above 8 years old and guardians of all the study participants in the prospective cohort signed informed consent forms to participate in the study. Signed consent forms were waived for the patients in the historical cohort. This study was approved by the Institutional Review Board of Seoul St. Mary’s hospital (KC18TESI0465).

### 2.5. Statistics

Categorical variables were compared by Pearson chi square test, and continuous variables were compared using Mann-Whitney U test. The binary logistic regression model was used to find the odds ratio for pathogen detection by cohort. A *p* value of <0.05 was considered statistically significant, and all tests were two sided.

## 3. Results

### 3.1. Study Population and Demographics

**Figure 1:**
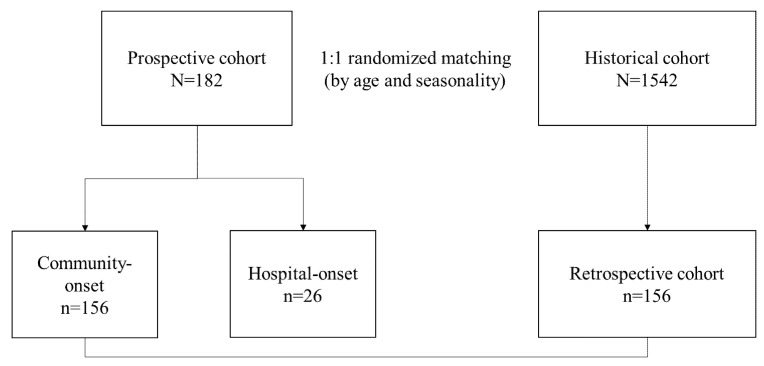
Flow chart of patients included in this study. A total of 214 patients with acute gastroenteritis were recruited. Patients that were immunocompromised or cases from the same patient that underwent stool studies were excluded (n = 32), and a final 182 patients were included in the prospective cohort.

During the 11-month period from 1 October 2019 to 31 August 2020, a total of 214 patients with acute diarrhea suspected of an infectious etiology were recruited. Patients that were immunocompromised or cases from the same patient that underwent stool studies were excluded (n = 32), and a final 182 patients were included in the prospective cohort. The median age of the patients was 3.8 (interquartile range (IQR), 0.9–8.2) years old, and 64.3% (n = 117/182) were male. The patients in the prospective cohort were divided into two groups depending on the onset of diarrhea: 85.7% (n = 156/182) had community-onset and 14.3% (n = 26/182) had hospital-onset (Figure 1).

Majority of the patients were recruited during the winter (56.0%, n = 102/182) and summer months (31.3%, n = 57/182). The overall positivity rate of the BioFire^®^ GI Panel in the prospective cohort was 57.1% (n = 104/182) (Table 1). This study was carried out in two parts: a prospective study recruiting children with new onset diarrhea during the prospective study period, and a retrospective cohort study of children diagnosed with acute infectious diarrhea during the 4 years prior to the prospective study period.

### 3.2. Comparison of Clinical Parameters between the Prospective and Historical Cohort with Community-Onset Diarrhea

A total of 156 patients with community-onset diarrhea in the prospective cohort were matched—by age and season—with 156 patients in the historical cohort with community-onset diarrhea to analyze the clinical utility of the BioFire^®^ GI Panel compared to conventional studies in the past (Supplementary Table S1). There were no significant differences in the age, gender, season of diagnosis, initial WBC count at admission, or initial CRP between the two groups (Table 2).

**Figure 2:**
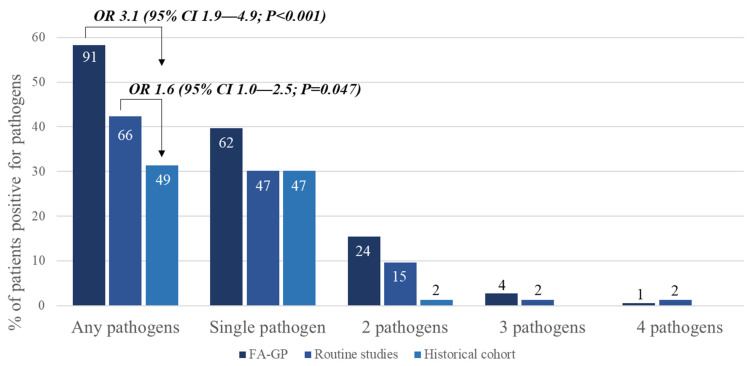
Pathogen positivity rate and number in the prospective and historical cohort. The odds ratio for a pathogen being detected in patients with acute diarrhea was 3.1 (95% confidence interval (CI) 1.9–4.9, *p* < 0.001) times higher in those with stool studies done by the FA-GP compared to those in the historical cohort. Numbers within the bars in the graph represent the actual number s of pathogens detected.

In the prospective group, the stool samples underwent BioFire^®^ GI Panel as well as conventional stool studies. There was a significantly higher pathogen positivity rate by the BioFire^®^ GI Panel (58.3%, n = 91/156) compared to routine stool studies (42.3%, n = 66/156) (*p* = 0.005) in the prospective group, and BioFire^®^ GI Panel compared to conventional stool studies done in the historical group (31.4%, n = 49/156) (*p* < 0.001). The odds ratio for a pathogen being detected in patients with acute diarrhea was 3.1 (95% confidence interval (CI) 1.9–4.9, *p* < 0.001) times higher in those with stool studies done by the BioFire^®^ GI Panel compared to those in the historical cohort (Figure 2).

Within the prospective cohort, 126 different pathogens from 91 stool samples were identified by the BioFire^®^ GI Panel and 51 from 49 stool specimens in the historical cohort, showing that the BioFire^®^ GI Panel had a higher detection of multiple pathogens from one stool sample (Figure 2). Of the detected pathogens, the BioFire^®^ GI Panel and historical cohort had similar percentages: bacteria (43.7% vs. 49.1%, *p* = 0.375), diarrheagenic *E. coli*/*Shigella* (21.4% vs. 15.1%, *p* = 0.385), and virus (34.9% vs. 32.1%, *p* = 0.841), respectively (Table 3).

**Figure 3:**
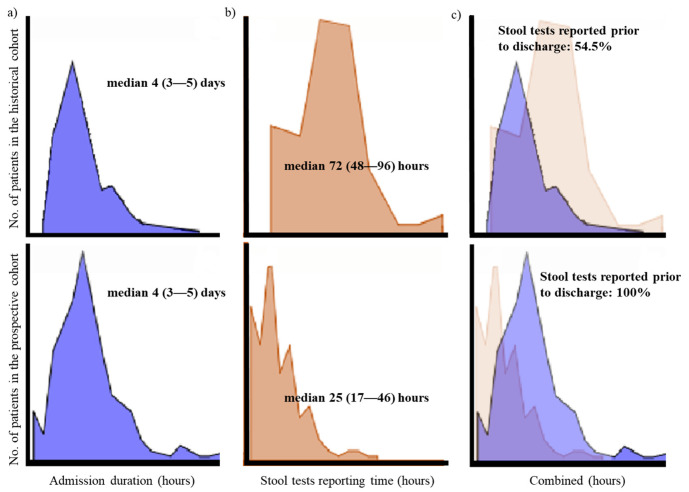
Comparison of admission duration and stool tests reporting between the historical vs. prospective cohort. (**a**) Although there was no significant difference in the admission duration of the patients in the historical vs. prospective cohort (*p* = 0.079), (**b**) the stool reporting time was significantly longer in the historical cohort compared to the prospective cohort (*p* < 0.001), (**c**) resulting in 45.5% of patients being discharged before receiving stool test results in the historical cohort.

The median admission duration in both the prospective cohort and historical cohort was 4 (IQR 3–5) days (*p* = 0.079). The stool tests reporting time after admission was 25 (IQR 17–46) hours for the BioFire^®^ GI Panel, and 72 (IQR 48–96) hours in the historical cohort (Table 2). Therefore, 100% of the patients in the prospective group were able to receive stool study results during admission, whereas 45.5% (n = 71/156) of the patients in the historical group were unable to receive their complete their stool test results prior to discharge (Figure 3).

A significantly lower percentage of patients in the prospective cohort that underwent stool testing with the BioFire^®^ GI Panel were administered antibiotics compared to patients in the historical cohort, 35.3% (n = 55/156) vs. 71.8% (n = 112/156) (*p* < 0.001), respectively.

### 3.3. Comparing BioFire^®^ GI Panel and Routine Conventional Studies in the Prospective Group with Community-Onset Diarrhea

In the prospective group, all 156 patients underwent both BioFire^®^ GI Panel and routine conventional studies (Supplementary Table S1). A total 56 pathogens with discrepancies between the two tests were identified. Pathogens that were detected by conventional studies but not detected by BioFire^®^ GI Panel made up 17.9% (n = 10/56) of the discrepancies. Of these, 50.0% (n = 5/10) were pathogens detected by stool cultures and were not included in the BioFire^®^ GI Panel. Furthermore, 82.1% (n = 46/56) of the pathogens detected by BioFire^®^ GI Panel were not detected by conventional studies. The most common pathogens detected by BioFire^®^ GI Panel alone were as follows: norovirus (n = 13, 23.2%), rotavirus (n = 8, 14.3%), and EPEC (n = 8, 14.3%) (Table 4).

### 3.4. Utility of the BioFire^®^ GI Panel on Infection Control and Prevention Actions

A total of 26 patients were recruited to undergo BioFire^®^ GI Panel and routine stool tests for hospital-onset acute diarrhea to aide in rapid decisions on precaution and isolation measures to prevent in-hospital infection transmission. A total of 53.8% (n = 14/26) of the patients with diarrhea onset after admission had one or more pathogens detected from their stools: 64.3% (n = 9/14) had a single pathogen detected, 28.6% (n = 4/14) had two pathogens detected, and 7.1% (n = 1/14) had three pathogens detected. Norovirus was the most common pathogen detected by the BioFire^®^ GI Panel (78.6%, n = 11/14), followed by *C. difficile* toxin A/B (35.7%, n = 5/14), and rotavirus (14.3%, n = 2/14).

Norovirus- or rotavirus-associated diarrhea require contact precaution and isolation infection control measures at the study center. Using the BioFire^®^ GI Panel, 38.5% (n = 5/13) of the patients with either rotavirus- or norovirus-associated diarrhea were isolated on the same day as diarrhea initiation, and 53.8% (n = 7/13) were isolated the very next day.

**Figure 4:**
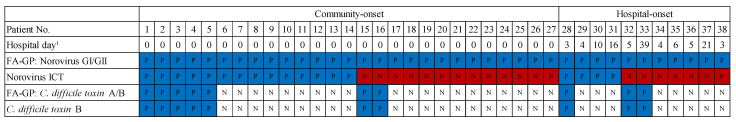
Detection of Norovirus and *C. difficile* toxin by FA-GP versus Norovirus ICT and C. difficile toxin B PCR in patients with community-onset and hospital-onset diarrhea. Compared to the FA-GP, norovirus ICT was only able to detect 14/27 (51.8%) patients with norovirus genes present in the stools of patients with community-onset diarrhea, and 4/11 (36.4%) in the stools of patients with hospital-onset diarrhea. However, the detection positivity was the same for FA-GP and AllplexTM GI-Bacteria(I) assay for *C. difficile* toxin. The P in shaded regions represent positive results, the N in white shaded regions represent negative results, and N in the red shaded regions represent negative result possibly leading to lack of infection prevention control measures. ^1^ Onset of diarrhea according to hospital day. FA-GP, FilmArray-gastrointestinal panel; ICT, immunochromatography test.

Compared to the BioFire^®^ GI Panel, norovirus ICT was only able to detect 4/11 (36.4%) patients with hospital-onset norovirus gastroenteritis, and 14/27 (51.8%) patients with community-onset diarrhea. Therefore, using only norovirus ICT for stool testing of norovirus would have caused a lack of infection control measures in 63.6% (n = 7/11) patients, leading to the possibility of in-hospital transmission at the pediatric ward. Furthermore, in patients admitted for community-onset norovirus gastroenteritis, 48.1% (n = 13/27) who were positive for norovirus detected by the BioFire^®^ GI Panel were negative by norovirus ICT. Therefore, these patients would have lacked contact precaution measures at admission due to false negative results (Figure 4) with the norovirus ICT alone.

Of the 24 *C. difficile* toxin genes identified, 19 from community-onset diarrhea specimens and 5 from hospital onset specimens, 10 (41.7%) were detected with norovirus. The detection positivity rate was the same for BioFire^®^ GI Panel and AllplexTM GI-Bacteria(I) assay for *C. difficile* toxin (Figure 4).

## 4. Discussion

Compared to conventional stool studies, the BioFire^®^ GI Panel rapidly and accurately detects a wide range of viruses, bacteria and parasites that can cause AGE [19,22,23]. With the introduction of the BioFire^®^ GI Panel, there have been controversies on the actual benefit and impact that the test may have in treating patients with AGE. However, studies related to the clinical utility of these tests have been lacking and real-world data on children with AGE are important and urgent [17,24]. To our knowledge, this study is the first prospective study on the clinical utility of BioFire^®^ GI Panel in pediatric AGE patients. This was a prospective study comparing the clinical utility of the BioFire^®^ GI Panel with conventional studies in a prospective and retrospective cohort. This study found a significantly higher pathogen positivity rate in the stools by the BioFire^®^ GI Panel (58.3%) compared with conventional stool studies (42.3%) and a historical group of children with AGE (31.4%). The stool tests reporting time after admission was 25 (IQR 17–46) hours for the BioFire^®^ GI Panel, and 72 (IQR 48–96) hours in the historical cohort. The rapid reporting time of the BioFire^®^ GI Panel led to a significantly lower percentage of patients being administered antibiotics for diarrhea, and 92.3% of the patients that required contact precaution and isolation were successfully given the needed measures within 48 h of diarrhea onset.

The main advantage of the BioFire^®^ GI Panel was that it was able to replace the five to six conventional stool testing methods available at the hospital, meanwhile maintaining high detection positivity rates for the 22 pathogens included in the panel. Like previous reports, the BioFire^®^ GI Panel showed a higher pathogen positivity rate than conventional stool tests in this study [17,25]. In fact, when BioFire^®^ GI Panel was compared to both conventional testing methods in the prospective and historical cohort, the detection positivity rates were significantly higher for both. The main discrepancies were found in viral pathogens, especially in the detection of norovirus and rotavirus for which only ICT methods were available. Additionally, pathogens that were not available for testing by conventional methods—such as adenovirus and astrovirus—were also detected (Table 4). We found in this study that compared to the historical cohort of children with AGE, rotavirus was detected at a lower incidence in the prospective cohort due to the decrease in rotaviral infections in accordance with the high rotavirus vaccine coverage in South Korea [26].

A statistically significant difference was also found in the stool test reporting time. By using the BioFire^®^ GI Panel, etiologic diagnosis could be confirmed at an average of 25 h after visiting the hospital. During working hours, the results of the BioFire^®^ GI Panel were reported within 4 h of stool collection. However, during non-working hours, patients needed to wait until the next working day to receive the results of the BioFire^®^ GI Panel. Nevertheless, the average stool reporting time was significantly shorter than conventional methods, where stool tests reporting time took an average of 72 h. Thus, all patients with stools tested by the BioFire^®^ GI Panel were able to receive either a positive or negative result within their admission period, however, only 54.5% of the patients in the historical cohort were able to receive all their results of the multiple stool tests prior to discharge. This meant that up to half the patients with conventional stool testing in the historical cohort were unable to incorporate the results of the stool tests into their treatment or intervention.

The rapid reporting time is another asset of the BioFire^®^ GI Panel. Although in this study, the rapid reporting time did not have any impact on the admission duration, there were two benefits of the BioFire^®^ GI Panel in clinical practice. The first was that the BioFire^®^ GI Panel was able to significantly reduce the use of antibiotics in patients with AGE. Since AGE often recovers without antibiotics, the routine use of antibiotics is not recommended before the causative agent is identified in uncomplicated AGEs in children [7,9]. However, it is also recommended that empiric antibiotics treatment can be administered if a clinician deems it necessary considering the clinical severity of the patient or epidemiological situations at the local regions [9]. Therefore, empirical antibiotics are often used in patients needing admission for moderate to severe AGE, leading to many instances where unnecessary and/or inappropriate antibiotics are given, as observed in the historical cohort of this study. In the prospective cohort, because clinicians were able to anticipate the rapid reporting time of the etiologic pathogen, the decision to administer antibiotics for moderate to severe diarrhea was made based on the etiologic pathogen detected by the BioFire^®^ GI Panel. This led to a decrease in the overall antibiotic usage. Furthermore, targeted antimicrobial therapy was possible with the BioFire^®^ GI Panel, which is particularly important in cases with STEC infections where the use of antibiotics may increase the incidence of hemolytic uremic syndrome [9]. Because it is difficult to completely discriminate STEC infections based on clinical symptoms or epidemiological conditions [27], the rapid confirmation to rule out or diagnose STEC through the BioFire^®^ GI Panel was convenient.

Second, the rapid reporting time of the BioFire^®^ GI Panel led to significant improvements in infection control and prevention actions. When hospital-onset acute diarrhea occurred, patients that had pathogens requiring infection precautions and isolation, such as norovirus and rotavirus, were quickly identified by the BioFire^®^ GI Panel. In this study, 92.3% of the patients that required contact precaution and isolation were successfully given the needed measures within 48 h of diarrhea onset. In addition, BioFire^®^ GI Panel showed a higher pathogen positivity rate compared to conventional studies for these pathogens, thereby reducing the likelihood of in-hospital transmission caused by false negative results. Because norovirus and rotavirus are pathogens with high transmissibility and have been the cause of hospital outbreaks, the utility of the BioFire^®^ GI Panel may be more advantageous than conventional studies in infection control and in hospital transmission due to the rapid reporting time and high detection sensitivities [11,28]. Furthermore, with the use of the BioFire^®^ GI Panel in pediatric emergency units especially during outbreaks such as the Coronavirus disease-19 (COVID-19) pandemic, the fast reporting time may be able to rule out more severe pathologies of diarrhea such as multisystem inflammatory syndrome, which may be a foreseen benefit and clinical value of the BioFire^®^ GI Panel.

In this study, the BioFire^®^ GI Panel showed a high percentage of patients with multiple pathogens detected, with percentages similar to previous studies [17,18,19,20]. The greater sensitivity of the BioFire^®^ GI Panel is thought to be the main reason for the higher pathogen positivity rate. Like other PCR methods, this test detects not only viable pathogens, but also non-viable pathogens and colonized pathobionts, which may cause difficulties for clinicians when managing the patient. One example from this study was *C. difficile*, which was detected frequently with norovirus. The percentage of asymptomatic colonization in healthy infants is high at 40–70%, and the association between diarrhea and *C. difficile* toxin in children under 2 years of age is poor [29,30,31]. Therefore, it is recommended that *C. difficile* tests be performed only when there are obvious risk factors or when *C. difficile* infection is strongly suspected for those under 3 years of age. Even after 3 years of age, it is recommended that children should be tested only when the risk factors for *C. difficile* infection, such as the history of taking antibiotics, are certain [32]. However, as multiplex-PCR-based gastrointestinal pathogen panels are increasingly used, the number of cases in which *C. difficile* is identified will also increase. It is therefore important for clinicians to cautiously interpret the results of the BioFire^®^ GI Panel for the pathogens detected and determine whether they are the cause of symptomatic infections or asymptomatic carriage.

With the use of BioFire^®^ GI Panel in clinical practice, clinicians are concerned about how far this multiplex-PCR-based GI pathogen panel test can replace conventional stool studies [23]. The comparative study showing discrepancies between the results of the BioFire^®^ GI Panel and routine conventional studies in the prospective cohort demonstrate that although the BioFire^®^ GI Panel showed high detection positivity rates of the pathogens included in the panel, 50.0% of the pathogens that were positive in the conventional studies and negative in the BioFire^®^ GI Panel were bacteria that were not included in the BioFire^®^ GI Panel but cultured from the stool. This included *Aeromonas* spp., *Y. frederiksenii*, and *Y. pseudotuberculosis*, of which both *Yersinia* spp. were clinically significant and important in patient management. The patient that had *Y. pseudotuberculosis* cultured presented with fever, rash, lymphadenopathy, conjunctival injection, diarrhea, and acute renal failure. This patient was initially misdiagnosed and treated for atypical Kawasaki disease, however was unresponsive to immunoglobulin therapy. When *Y. pseudotuberculosis* was cultured from the stool, the patient was treated with antibiotics and improved. This was another limitation of the BioFire^®^ GI Panel, highlighting the importance of stool cultures which are gold standard in the pathogenic diagnoses of infectious AGE, and draws attention to the fact that BioFire^®^ GI Panel may not be able to replace stool cultures, which are also important for antibiotic susceptibility testing of pathogens.

The unique advantage of this study is that the prospective cohort and retrospective historical cohorts were matched 1:1 to analyze the clinical utility of the BioFire^®^ GI Panel. Additionally, patients in the prospective cohort underwent stool testing with both the BioFire^®^ GI Panel and conventional stool studies to compare positivity rates, discrepancies, and how these factored into patient care. However, because the control group was a historical cohort of patients, it was difficult to remove selection bias.

Despite these limitations, through this prospective study, it was possible to observe that the high positivity rate and rapid reporting time of the BioFire^®^ GI Panel had clinical benefits for children admitted for acute diarrhea, especially by significantly reducing the antibiotic misuse and enabling early adequate infection precaution and isolation. With the increasing availability of multiplex-PCR-based gastrointestinal pathogen panels, more research is needed to utilize this test to provide better clinical management for patients.

## Figures and Tables

**Table 1:** Demographics and pathogen detection of patients in the prospective cohort.

	N = 182
Median age, years (IQR)	3.8 (0.9–8.2)
Sex, male	117 (64.3%)
Underlying disease	
None	177 (97.3%)
Congenital heart disease	1 (0.5%)
Feeding disorder	1 (0.5%)
Neurologic disease	2 (1.1%)
Hemato-oncologic malignancy	1 (0.5%)
Onset of diarrhea	
Community-onset	156 (85.7%)
Hospital-onset	26 (14.3%)
Diagnosed season	
Spring	12 (6.6%)
Summer	57 (31.3%)
Fall	11 (6.0%)
Winter	102 (56.0%)
Pathogen detection	
Any pathogen	104 (57.1%)
Single pathogen	71 (39.0%)
2 pathogens	27 (14.8%)
3 pathogens	6 (3.3%)

IQR, interquartile range; Spring, March-May; Summer, June-August; Fall, September-November; Winter, December-February.

**Table 2:** Comparison of clinical parameters between the prospective and historical cohorts.

	Prospective n = 156	Historical n = 156	*p*
Age, years, median (IQR)	4.8 (1.2–9.0)	4.2 (1.0–8.5)	0.328
Male, n (%)	98 (62.8)	95 (61.0)	0.727
No. of patients admitted, n (%)	149 (95.5)	151 (96.8)	0.556
Season of diagnosis			1.000
Spring, n (%)	10 (6.4)	10 (6.4)	
Summer, n (%)	55 (35.3)	55 (35.3)	
Fall, n (%)	10 (6.4)	10 (6.4)	
Winter, n (%)	81 (51.9)	81 (51.9)	
WBC, 10^6^/L, median (IQR)	9110 (6310–12,800)	9860 (7403–12,918)	0.124
CRP, mg/dL, median (IQR)	2.3 (0.2–5.5)	1.68 (0.32–5.71)	0.596
Admission duration, days, median (IQR)	4 (3–5)	4 (3–5)	0.079
Stool tests reporting time, hours, median (IQR)	25 (17–46)	72 (48–96)	<0.001

Spring, March-May; Summer, June-August; Fall, September-November; Winter, December-February.

**Table 3:** Pathogens detected in prospective cohort by BioFire^®^ GI Panel and historical cohort with community-onset diarrhea.

		No. of Pathogens (%)		*p*
		BioFire^®^ GI Panel (n = 126)	Historical (n = 51)	
Bacteria	*Aeromonas* spp.			
	*Campylobacter* spp.	21 (16.7)	10 (18.9)	
	*Clostridium difficile* toxin	19 (15.1)	4 (7.5)	
	*Plesiomonas shigelloides*	1 (0.8)	-	
	*Salmonella* spp.	8 (6.3)	12 (22.6)	
	*Yersinia enterocolitica*	6 (4.8)	-	
	*Yersinia frederiksenii*			
	*Yersinia pseudotuberculosis*			
	Subtotal	55 (43.7)	26 (49.1)	0.375
Diarrheagenic *E. coli*/*Shigella*	EAEC	1 (0.8)	4 (7.5)	
	EIEC	1 (0.8)	-	
	EPEC	20 (15.9)	3 (5.7)	
	ETEC	1 (0.8)	1 (1.9)	
	STEC	4 (3.2)	-	
	Subtotal	27 (21.4)	8 (15.1)	0.385
Virus	Adenovirus	2 (1.6)	-	
	Astrovirus	2 (1.6)	-	
	Norovirus	27 (21.4)	5 (9.4)	
	Rotavirus	10 (0.8)	12 (22.6)	
	Sapovirus	3 (2.4)	-	
	Subtotal	44 (34.9)	17 (32.1)	0.841

EAEC, Enteroaggregative *E. coli*; EIEC, Shigella/Enteroinvasive *E. coli*; EPEC, Enteropathogenic *E. coli*; ETEC, Enterotoxigenic *E. coli*; STEC, Shiga-like toxin-producing *E. coli*.

**Table 4:** Discrepancies of identified stool pathogens in BioFire^®^ GI Panel and conventional tests in the prospective cohort with community-onset diarrhea.

BioFire^®^ GI Panel	Routine Test	No. of Cases (%) N = 56
Negative	*Aeromonas* spp.	3 (5.4)
Negative	*Salmonella* spp.	1 (1.8)
Negative	EAEC	2 (3.6)
Negative	*Y. frederiksenii*	1 (1.8)
Negative	*Y. pseudotuberculosis*	1 (1.8)
Negative	*Rotavirus*	2 (3.6)
	Subtotal	10 (17.9)
*Clostridium difficile* toxin	Negative	3 (5.4)
*Plesiomonas shigelloides*	Negative	1 (1.8)
*Salmonella* spp.	Negative	2 (3.6)
EAEC	Negative	1 (1.8)
EIEC	Negative	1 (1.8)
EPEC	Negative	8 (14.3)
STEC	Negative	2 (3.6)
Adenovirus	Negative	2 (3.6)
Astrovirus	Negative	2 (3.6)
Norovirus	Negative	13 (23.2)
Rotavirus	Negative	8 (14.3)
Sapovirus	Negative	3 (5.4)
	Subtotal	46 (82.1)

## Data Availability

The data presented in this study are available on request from the corresponding author. The data are not publicly available due to ethical reasons.

## Supplementary Materials

The following are available online at https://www.mdpi.com/article/10.3390/diagnostics11071175/s1, Table S1: Methods to detect BioFire^®^ FilmArray^®^ Gastrointestinal Panel pathogens by routine studies in the prospective and historical cohorts, Table S2: Intervention protocol by pathogens detected.
