# Supplementary material for: Quality Improvements in Management of Children with Acute Diarrhea Using a Multiplex-PCR-Based Gastrointestinal Pathogen Panel
Source: Diagnostics (Basel). 2021 Jun 28;11(7):1175. doi: 10.3390/diagnostics11071175 (PMC8303787; doi:10.3390/diagnostics11071175)
Supplement: Supplementary file 1 [file diagnostics-11-01175-s001.zip › diagnostics-1265892-supplementary.pdf]

Supplementary

Clinical Utility of Film Array Gastrointestinal Panel in Children with Acute diarrhea

Authors and Affiliations

In Hyuk Yoo[1], Hyun Mi Kang[1*], Woosuk Suh[2], Hanwool Cho[3], In Young Yoo[3], Sung Jin Jo[3], Yeon-Joon Park[3], and Dae Chul Jeong[1*]

[1]Department of Pediatrics, College of Medicine, The Catholic University of Korea, Seoul, Korea

[2]Department of Pediatrics, Uijeongbu Eulji Medical Center, Eulji University School of Medicine

[3]Department of Laboratory Medicine, College of Medicine, The Catholic University of Korea, Seoul, Korea

*These authors should be considered co-senior authors.

Table S1. Methods to detect BioFire® FilmArray® Gastrointestinal Panel pathogens by routine studies in the prospective and historical cohorts

Prospective cohort		Historical cohort
BioFire® FilmArray® GI Panel	Routine Stool tests	Routine Stool tests
**Bacteria**		
Campylobacter	Culture, PCR	Culture
Clostridium difficile (Toxin A/B)	Culture, toxin PCR	Culture, toxin PCR
Plesiomonas shigelloides	Culture	Culture
Salmonella	Culture, PCR	Culture, PCR
Vibrio	Culture, PCR	Culture
Vibrio cholerae	Culture	Culture
Yersinia enterocolitica	Culture, PCR	Culture
**Diarrheagenic E. coli/Shigella**		
E. coli O157	PCR, Culture	PCR, Culture
EAEC	PCR	PCR
EIEC	PCR	PCR
EPEC	PCR	PCR
ETEC	PCR	PCR
STEC	PCR	PCR
**Virus**		
Adenovirus F 40/41	None	None
Astrovirus	None	None
Norovirus GI/GII	ICT	ICT
Rotavirus A	ICT	ICT
Sapovirus	None	None
**Protozoa**		
Cryptosporidium	Microscopy	Microscopy
Cyclospora cayetanensis	Microscopy	Microscopy
Entamoeba histolytica	Microscopy	Microscopy
Giardia lamblia	Microscopy	Microscopy

EAEC, Enteroaggregative *E. coli*; EIEC, Shigella/ Enteroinvasive *E. coli*; EPEC, Enteropathogenic *E. coli*; ETEC, Enterotoxigenic *E. coli*; ICT, Immunochromatography test; PCR, polymerase chain reaction; STEC, Shiga-like toxin-producing *E. coli*;

Table S2. Intervention protocol by pathogens detected.

Detected Pathogen	Intervention based on Stool test results	
	Antibiotics	Infection control
**Bacteria**		
Aeromonas	None	SP
Campylobacter	Azithromycin	SP
Clostridium difficile (Toxin A/B)	Metronidazole	CP
Plesiomonas shigelloides	3rd G cephalospoin	SP
Salmonella	3rd G cephalospoin	CP
Vibrio	None	CP
Vibrio cholerae	Azithromycin	CP
Yersinia enterocolitica	TMP/SMX	CP
**Diarrheagenic E. coli/Shigella**		
E. coli O157	None	CP
EAEC	3rd G cephalospoin	CP
EIEC	3rd G cephalospoin	CP
EPEC	3rd G cephalospoin	CP
ETEC	3rd G cephalospoin	CP
STEC	None	CP
**Virus**		
Adenovirus F 40/41	None	CP
Astrovirus	None	CP
Norovirus GI/GII	None	CP & isolation
Rotavirus A	None	CP & isolation
Sapovirus	None	CP
**Protozoa**		
Cryptosporidium	Azithromycin	CP
Cyclospora cayetanensis	TMP/SMX	CP
Entamoeba histolytica	Metronidazole	CP
Giardia lamblia	Metronidazole	CP

3rd G, third generation; CP, contact precaution; EAEC, Enteroaggregative *E. coli*; EIEC, Shigella/ Enteroinvasive *E. coli*; EPEC, Enteropathogenic *E. coli*; ETEC, Enterotoxigenic *E. coli*; SP, standard precaution; STEC, Shiga-like toxin-producing *E. coli*;
